# Do I decide my career? Linking career stress, career exploration, and future work self to career planning or indecision

**DOI:** 10.3389/fpsyg.2022.997984

**Published:** 2022-08-23

**Authors:** Zemei Zhang, Xuan Yu, Xuhong Liu

**Affiliations:** ^1^Business School, Sichuan University, Chengdu, China; ^2^School of Economics and Management, Southwest Petroleum University, Chengdu, China; ^3^Department of Police Management, Sichuan Police College, Luzhou, China

**Keywords:** career stress, career exploration, future work self, career planning, career indecision

## Abstract

Career planning and career decision are important tasks for college students. The process may be affected by career stress, career exploration, and future work self, with some students facing career indecision. Combining both construction career and proactive motivation model, this study investigated the relationships between career stress, career exploration, future work self, career planning and career indecision among 1,012 Chinese college students using the Structure equation model (SEM) to test the proposed mediation model. Results show that career stress negatively predicts career planning, while career exploration positively predicts career planning. The mediating role of future work self has on career stress and career exploration's effects on both career planning and career indecision was verified. Moreover, the study also found future work self's positive effects on both career planning and career indecision in Chinese college students. Finally, the study's theoretical and practical significance and implications are discussed.

## Introduction

Finding career prospects has come into an era knows as VUCA or volatility, uncertainty, complexity and ambiguity where career construction becomes an important task. Career construction theory (Savickas, 2021) posits that a career is a psychological construct created across an entire life span, and focuses the career seeker's attention on self-reflecting on their actions and experiences. Since there is nothing in mind that is not first of all in society (Vygotsky, 1978), career construction is an interactive process between individual and society (Savickas, 2021). This study focuses on the role of external situational (such as career stress) and individual factors (such as career exploration) in the construction of college students' career and explores the specific results on career planning and career indecision.

Career planning is an important task for college students—this includes setting career goals, developing plans and formulating strategies for a career (Gould, 1979; Renn et al., 2014). Studies have shown that career planning is related to higher career effectiveness (Gould, 1979), ensures the quality of re-employment (Zikic and Klehe, 2006), and helps realizing career calling (Yuliawati and Ardyan, 2022). But not everyone has a career plan because some students will face career indecision. Career indecision is defined as the inability to make an occupational or educational decision when asked to do so, and delays bringing closure of the career decision making process (Osipow, 1999). According to the developmental perspective, career indecision is a necessary but transitory step that precedes career decision-making, which gradually decreases over time (Vignoli, 2015). However, some people may experience long-term career decision-making difficulties due to internal and external factors (Argyropoulou et al., 2007; Saka and Gati, 2007). Likewise, for Chinese college students, career indecision remains as an urgent problem to be solved in career counseling.

Career stress has an important effect on college students' career construction. According to “The white Paper” on the growth of Chinese college students in 2017, a survey on the perplexing themes and solutions for students through nearly 80,000 college students from 653 schools, the 10 current most perplexing themes are the following: learning, career organization, daily practice, self-management, emotion, spirit, and value worry about the future. Most students suffer from great career stress. Limited studies have focused on career stress among Chinese students where most only consider employment stress. For example, Sun et al. (2021) discussed the negative impact of college students' employment stress on positive psychological capital and delayed career satisfaction. Although employment stress may represent a part of college students' career stress, it does not represent all of it. (Boo and Kim, 2020) proposed that students who are unprepared for their future careers face greater stress and lead to career indecision. Choi et al. (2011) suggested that career stress faced by college students also includes occupational ambiguity, external conflicts, and lack of information. From a career construction perspective, the deeper problem that needs to be solved is clarity of career identity (Erikson, 1968; Arnett, 2000). This means students need to figure out what they want to do in the future, how to handle conflicts with important others and so on, but is more a matter of career pressure than merely employment stress. Previous studies have proved that career stress is indeed related to career indecision (Choi et al., 2011; Boo and Kim, 2020), but the specific mechanism remains unexplored.

According to Savickas (2021), the self evolves in activity as it internalizes cultural and social practices from its external world. Likewise, Career exploration is an important activity for college students which helps them learn career-related knowledge through interaction with their external environment (Super, 1957). A large number of studies have proved the importance of career exploration for college students' career development such as enhancing their perception of individual internal characteristics, gaining knowledge about education and career choices, and facilitating career planning (Jordaan, 1963; Blustein, 1997; Zikic and Hall, 2009). However, there remains a great controversy on the role of career exploration in career indecision (Jiang et al., 2019).

Overall, previous studies concern either career stress or career exploration among college students, but lack considering both together. There remains some debates about the effects (Jiang et al., 2019), and the influence mechanism needs further exploration. The current study is supplemented in the following aspects: first, considering the specific stage of college students, we explore both career stress and career exploration's effects on career construction. Second, combining both career construction theory (Savickas, 2021) and proactive motivation model (Parker et al., 2010), we explore the role of the future work self. Career construction theory emphasizes self-construction only within the influence of internal and external factors. Hence, future work self (Strauss et al., 2012) represents a hoped-for possible self in relation to work, and may be affected by external situational (such as career stress) and individual factors (such as career exploration). According to proactive motivation model, future work self captures the self-starting motive (“reason to”) to pursue desirable future career possibilities, thereby stimulating individuals to engage in various proactive career behaviors, such as career planning, skill development and so on (Strauss et al., 2012). Thus, future work self can also be considered as the motivational factor for career planning and decision (Taber and Blankemeyer, 2015). Therefore, we propose that future work self plays a mediating role in the effects of career stress and career exploration on both career planning and career indecision.

This study offers three contributions to the current career research literature. First, we consider both career stress and career exploration, which is more relevant to the students' realistic situation. Secondly, based on career construction theory, we combine subjective initiative with career context to explore the joint effects, thereby expanding the application of career construction theory. Finally, we extend the stream of existing literature by targeting self-image for the future job (i.e., future work self) as a mechanism that connects career stress and career exploration with career plans and career indecision, while bringing some enlightenment to career stress management and career counseling.

## Theory and hypothesis development

### Career stress, career exploration and future work self

Career stress refers to the stress related to occupation problems. These include factors such as occupational ambiguity, lack of information, employment pressure and external conflicts (Choi et al., 2011). Commonly encountered by college students, studies have demonstrated its negative effects. In stressful situations, the interaction between individuals and their environment triggers a series of physiological and psychological reactions such as tension and anxiety (Lazarus and Folkman, 1984), which are also often accompanied by reduced self-efficacy and career decision-making difficulties (Kelly and Lee, 2002; Vignoli, 2015; Park et al., 2017). Moreover, coping with stress is a process of resource consumption (Lazarus and Folkman, 1984). Generally, studies skew toward the negative effects of occupational stress.

Career exploration is a process characterized by exploratory behaviors and cognitions related to vocational development (Stumpf et al., 1983). Career exploration was once a major task in adolescence and early adulthood (Super, 1957; Jordaan, 1963). With the development of boundaryless careers, it has instead been regarded as a lifelong career task (Zikic and Hall, 2009). However, college students' career exploration remains a severe test due to the interwoven effects of various factors emerging in adulthood (Arnett, 2000). Career exploration includes self-exploration and environment exploration (Stumpf et al., 1983) and is an important channel for college students to obtain career opportunities and resources (Flum and Blustein, 2000; Savickas, 2002). Studies have shown that career exploration contributes to increasing young people's knowledge of themselves and the working world (Cheung and Arnold, 2014; Cheung and Jin, 2016; Lent et al., 2017), their achievement of career goals (Hu et al., 200), and achievement of personal identity (Zikic and Klehe, 2006; Zikic and Hall, 2009).

According to the career construction theory, the individual constructs itself through a process of interaction between its external and internal factors, subsequently leading to the construction of one's own career. Therefore, self-construction can be seen as a core task for college students affected by career stress and career exploration. Career stress can therefore be seen as an external factor, while career exploration is seen as an internal one. The salience of future work self can be seen as a kind of self-construction—the future work self is derived from the possible conception of the self and is hereby defined as an image or a reflection of an individual in future work expectations and ambitions (Strauss et al., 2012). It is both the embodiment and extension of the individual's “possible self” in the workplace (Markus and Nurius, 1986).

Studies have found that the future work self, as a future-oriented self-concept, is influenced by both occupational behavior and context. Savickas (2002) suggests that occupational behaviors can help individuals accumulate adaptative resources and rebuild their cognition of future occupational images. Both Guan et al. (2017) and Xiao et al. (2021) proved that career exploration has a positive effect on college students' future work selves. Contexts also play an important role—Kao et al. (2020) studied the supportive context of future work self and found that social psychological guidance is conducive to the improvement of the salience of future work self, thus affecting the individual's behavior job-hunting. Given its negative effects on career behaviors, career stress may have negative effects on the silence of the future work self. We therefore hypothesize the following:

**Hypothesis 1: career stress negatively predicts future work self; and**

**Hypothesis 2: career exploration positively predicts future work self**.

### Future work self and career planning, career indecision

Proactive motivation model (Parker et al., 2010) concerns the origin of proactive goal generation and striving, and suggests that there are common motivational processes across different types of proactive behavior, one of which is “reason to” motivation. Future work self provides the “reason to” motivation because it captures an individual's hopes and aspirations in relation to his or her career and constitutes motivational resources that individuals can use in the control and direction of their own actions (Parker et al., 2010). The motivational role therefore of the future work self is thereby reflected in three aspects (Strauss et al., 2012): first, the future work self makes people aware of the discrepancy between the current self and the imagined ideal future work self, which then enables people to work toward an imagined future. Second, the future work self allows the individual a more playful and exploratory approach to redefine themselves, which potentially stretches the individual's aspirations and broadens their creative thinking about future possibilities. Third, the future work self-initiates the process of mental simulation which refers to the “projection of the self into the future,” making people aware of the gap between present abilities and anticipated future demand, thus motivating future oriented behaviors. Studies have found that the future work self, as a kind of motivation, encourages individuals to pursue their ideal future career possibilities (Strauss et al., 2012), and affects individual career attitudes and behaviors such as proactive career behavior (Taber and Blankemeyer, 2015), career adaptability (Guan et al., 2017), job hunting behavior (Kao et al., 2020), and job performance (Lin et al., 2016). Therefore, a salient future work self will help individual actively engage in their own career planning and reduce career indecision.

Gould (1979) found that individuals with higher identity resolution reported more extensive career planning, implying that individuals with a clear future identity may likewise have a clear career plan. Taber and Blankemeyer (2015) found that salient future work self predicts career planning. Chishima and Wilson (2020) used a letter-exchange exercise to enhance students' future self-continuity and further proved the relationship between future self-continuity and academic or career planning for high school students.

Gati et al. (1996) proposed that lacking motivation can conversely lead to career indecision. Saka and Gati (2007) classified both self and identity-related variables as major clusters correlated with career indecision. Studies have shown that those with moratorium and diffused identity had more career decision-making difficulties (Blustein et al., 1991; Nauta and Kahn, 2007). Considering the motivational role of the future work self, those with salient versions of it may less likely face career indecision. We therefore hypothesize the following:

**Hypothesis 3: future work self positively predicts career planning; and**

**Hypothesis 4: future work self negatively predicts career indecision**.

### Mediating role of future work self

It is common for college students to make career planning and career decisions under career stress where career exploration also plays a key role. Recently, while various studies have looked into the direct effects of career stress and career exploration on career planning and career indecision, only a few have considered its influencing mechanism. Studies found that career stress may have negative effects on students' career plans and decisions (Saunders et al., 2000; Choi et al., 2011; Cho and Lee, 2017) while career exploration may help establish coherent ones (Zikic and Klehe, 2006) although it remains controversial if career exploration does, indeed, reduce career indecision. While some studies have found that career exploration can help manage stress related to career indecision and ultimately reduce it (Xu et al., 2014; Praskova et al., 2015; Park et al., 2017), other studies found that career exploration provides otherwise. Downing and Nauta (2010) found that young adults at university become more indecisive the more they explore their careers.

Further exploration of the influence mechanism is needed. According to career construction theory, there exist necessary steps to carving out a career path (Savickas, 2021). When individuals reach late adolescence, they gradually integrate their actions and agency into a unique identity under the influence of internal and external factors. The resulting identity and its successive revisions will impose meaning on vocational behavior to construct one's unique career. It can be implied that under the combined functions of career stress and career exploration, college students gradually clarify their expectations for the future, thus make their own career plans and decisions. Career exploration allows them to accumulate resources to cope with career stress and develop their own values and attitudes linked to a self-image in a future work domain (Zikic and Klehe, 2006), and to establish one's future work self. According to proactive motivation model (Parker et al., 2010), future work self also can be seen as a motivational factor that influences students' vocational behavior (Strauss et al., 2012). Hence, the more salient one's future work self is, the clearer their career plans may become and the less career indecision may be faced.

Summarily, combining both career development theory and proactive motivation model, the future work self may play a mediating role and is the result of both the interaction between situation and individual and the motivational factor of career behaviors (Markus and Nurius, 1986; Guan et al., 2017). Two studies have proved the mediating effect of future work self: Xiao et al. (2021) proved that the future work self plays a mediating role in college students' career exploration and adaptability. Meanwhile, Kao et al. (2020) proved that future work selves mediate the relationship between psychosocial mentoring and job search behaviors of college students. Thus, we hypothesize the following:

**Hypothesis 5: the future work self plays a mediating role in the relationship between career stress and (a) career planning and (b) career indecision; and**

**Hypothesis 6: the future work self plays a mediating role in the relationship between career exploration and (a) career planning and (b) career indecision**.

## Materials and methods

In order to clarify how Chinese college students construct their careers in the context of career stress and how career exploration affects in this process, combining career construction theory and proactive motivation model, this research conducted an empirical study on the relationship between career stress, career exploration, future work self, career plan and career indecision. The effects of career stress and career exploration on future work self, career planning and career indecision, as well as the mediating effect of future work self were tested.

### Participants and procedures

A cluster random sampling method was used to select 1,077 students from three colleges in Chongqing, China, in 2021, including Chongqing Technology and Business University, Chongqing Normal University and Chongqing University of Technology. We selected different categories of departments and majors within the three colleges, contacted with the counselors of various majors, informed them of our research purpose, and asked them to appoint class representatives for us to answer our questionnaires. The data collection is relatively comprehensive. To prevent the deviation of homologous methods and improve the data validity, career pressure, career exploration, future work self, career planning, and career indecision was measured at various time points over a 3-week period. At the first time point (T1), data about respondents' personal information were collected and career stress and career exploration were measured in turn. During the second time point (T2) a week later, their future work selves were measured. Finally, the third time point (T3) saw both career planning and career indecision being measured. Moreover, this research has set up the last four digits of the respondents' mobile phone numbers separately so that the data corresponding to the abovementioned variables could be effectively matched.

SPSS was used to analyze and process the data while descriptive statistics and correlation analysis were conducted on participants' background information, career stress, career exploration, and other variables. Next, simple regression analysis and multiple linear regression analysis were used to test the relationship between career stress, career exploration and career planning, career indecision. Finally, structural equation model (SEM) was used to test the proposed mediation model.

A total of 1,077 complete questionnaires were obtained by matching the last four digits of the mobile phone number. After incomplete questionnaires had been excluded, 1,012 valid questionnaires (93.96%) were collected. The characteristics of sample data are shown in Table 1. In the statistics of these demographic variables, for gender variable, 1 = male, 2 = female; For age variable, 1 = under 18, 2 = between 19 and 20, 3 = 21 and above; For students' year of study variable, 1 = freshmen, 2 = sophomores, 3 = juniors, 4 = seniors; For major variable, 1 = science, 2 = management, 3 = economics, 4 = engineering; 5 = law; 6 = education; 7 = literature; 8= history.

**Table 1:** Demographic information of participants (*N* = 1,012).

**Background characteristics**		***N***	**Proportion (%)**
Gender	Male	228	22.5
	Female	784	77.5
Age	Under 18	102	10.1
	Between 19 and 20	601	59.4
	21 and above	309	30.5
Students' year of study	Freshmen	463	45.8
	Sophomores	235	23.2
	Juniors	286	28.3
	Seniors	28	2.8
Major	Science	59	5.8
	Management	651	64.3
	Economics	158	15.6
	Engineering	107	10.6
	Law	28	2.8
	Education	4	0.4
	Literature	4	0.4
	History	1	0.1

### Measurements

This study adopted mature Western scales to measure the variables. To ensure the consistency and applicability of the English scale in the Chinese context, the author conducted a translation-back-translation procedure (Brislin, 1986). Before formal investigation, we conducted a preliminary test on 15 college students and modified some items according to their feedback.

Career stress was measured with the scale developed by Choi et al. (2011). Responses were on a five-point Likert scale ranging from 1 (strongly disagree) to 5 (strongly agree) which included statements such as “I feel frustrated because not a lot of people are helping me prepare for my career.” The Cronbach's alpha for this scale was at 0.946.

Career exploration was measured with the scale developed by Stumpf et al. (1983). Participants were asked the extent they have behaved in the following ways the past 3 months. Responses were on a five-point Likert scale ranging from 1 (strongly disagree) to 5 (strongly agree) which included statements such as “I obtained information on the labor market and general job opportunities in my career area.” Cronbach's alpha for this scale was at 0.911.

Future work self was measured with the scale developed by Strauss et al. (2012) and consisted of five items which included statements such as “This future is very easy for me to imagine.” Following the procedure proposed by Strauss et al. (2012), respondents were asked to imagine their ideal future self in relation to their job, remember these mental images, and were then asked to rate these mental images on a five-point Likert scale ranging from 1 (strongly disagree) to 5 (strongly agree). Cronbach's alpha for this scale was 0.895.

Career planning was measured using the scale developed by Gould (1979) and consisted of six items. Responses were on a six-point Likert scale ranging from 1 (strongly disagree) to 6 (strongly agree) which included statements such as “I have a strategy for achieving my career goals.” The Cronbach's alpha for this scale was at 0.941.

Career indecision was measured using Jones and Lohmann's (1998) three dimensions of Career Decision Profile: decidedness, career choice importance, and knowledge about occupations and training. We did not distinguish the dimensions of career indecision in our study, and instead took them as a whole indicator. Responses were on a eight-point Likert scale ranging from 1 (strongly disagree) to 8 (strongly agree) which included statements such as “I know what my interests and abilities are, but I am unsure how to find occupations that match them.” The Cronbach's alpha for this scale was at 0.730.

Control variables were selected based on previous studies related to career planning and career indecision and included age, gender, students' year of study, major, career decision self-efficacy and positive affect. First, to exclude the potential bias effect of demographic variables on the relationship, we took age, gender, students' year of study and major as control variables of our model. Second, according to the proactive motivation model, “can do” factors such as career decision self-efficacy and “energized to” factors such as positive affect also serve as important driving forces for individuals' proactive career behavior. The role of career decision self-efficacy in influencing career behavior has been confirmed in many studies like Guay et al. (2003) and Choi et al. (2012). The effect of positive affect on career behavior was also confirmed in studies conducted by Bindl and Parker (2010) and Park et al. (2018). So we use career decision self-efficacy and positive affect as control variables. Career decision self-efficacy was measured using CEDSE-BD (Brief Decisional Self-Efficacy Factor), a subscale of Career Exploration and Decision Self-Efficacy Scale (CEDSE) developed by Lent et al. (2016), which included items such as “How much confidence do you have in your ability to identify careers that best use your skills.” Participants were asked to rate their level of confidence on a 10-point scale, from 1 (no confidence at all) to 10 (complete confidence). The Cronbach's alpha for this scale was at 0.964. Positive affect was measured using scales devised by Warr (1990) to measure the affective dimensions tense—calm and depressed—enthusiastic. Respondents were asked to think about the past few weeks and indicate to what extent they had been in the state indicated by the items. The items on the tense—calm scale were “tense,” “uneasy,” “worried,” “calm,” “contented” and “relaxed”. Those on the depressed—enthusiastic scale were “depressed,” “gloomy,” “miserable,” “cheerful,” “optimistic,” “enthusiastic”. Responses were on a seven-point Likert scale ranging from 1 (never) to 6 (all of the time). Scores on the items that tapped the negative poles were reversed, so that the total score on each scale represented the level of positive affect in the corresponding affective dimension. The Cronbach's alpha for this scale was at 0.770.

## Results

Table 2 presents the correlations for the study variables.

**Table 2:** Correlations of study variables.

**Variables**	**1**	**2**	**3**	**4**	**5**
1. Career stress	1				
2. Career exploration	−0.062*	1			
3. Future work self	−0.125**	0.424**	1		
4. Career indecision	0.142**	0.154**	0.211**	1	
5. Career planning	−0.179**	0.511**	0.504**	0.192**	
6. Gender	0.012	−0.099**	−0.088**	−0.080*	−0.053
7. Age	0.062*	−0.019	−0.019	−0.049	−0.026
8. Students' year of study	0.088**	0.025	−0.059	−0.057	−0.034
9. Major	−0.006	−0.063*	−0.036	−0.014	0.051
10. Career decision self-efficacy	−0.173**	0.425**	0.429**	0.254**	0.455**
11. Positive affect	0.097**	0.226**	0.239**	0.303**	0.186**

N = 1,012.

*p < 0.05, **p < 0.01.

AMOS 24.0 was used for confirmatory factor analysis. The alpha coefficient of career stress scale was at 0.946. AVE = 0.677, CR = 0.943. The alpha coefficient of career exploration scale was at 0.911. AVE = 0.684, CR = 0.906. The alpha coefficient of future work self scale was at 0.895. AVE = 0.798, CR = 0.897. The alpha coefficient of the career indecision scale was at 0.730. AVE = 0.532, CR = 0.706. The alpha coefficient of career planning scale was at 0.941. AVE = 0.857, CR = 0.943.

Table 3 presents the confirmatory factor analysis index results showed that the five-factor models were all within acceptable range and were significantly better than other models (χ2/df = 3.450, RMSEA = 0.049, SRMR = 0.076, CFI = 0.916, TLI = 0.909, IFI = 0.917), indicating that all variables have good discriminant validity.

**Table 3:** Confirmatory factor analysis index results.

**Model**	***χ2/df***	**RMSEA**	**SRMR**	**CFI**	**TLI**	**IFI**
1. Five-factor (CS, CE, FWS, CI, CP)	3.450	0.049	0.076	0.916	0.909	0.917
2. Four-factor (CS + CE, FWS, CI, CP)	4.105	0.055	0.112	0.893	0.885	0.895
3. Four-factor (CS, FWS, CE + CP, CI)	4.747	0.061	0.134	0.870	0.862	0.871
4. Four-factor (CS + CI, FWS, CE, CP)	4.837	0.062	0.131	0.867	0.858	0.868
5. Three-factor (CS + CI + CP, FWS, CE)	5.171	0.064	0.247	0.855	0.846	0.856
6. Three-factor (CS + CE + FWS, CI, CP)	5.313	0.065	0.320	0.850	0.841	0.851
7. Two-factor (CS + CE + FWS + CI, CP)	5.303	0.065	0.327	0.850	0.841	0.851
8. Two-factor (CS + CE + CI + CP, FWS)	5.331	0.065	0.325	0.850	0.840	0.850
9. One-factor (CS + CE + FWS + CI + CP)	5.373	0.066	0.340	0.848	0.838	0.848

CS, career stress; CE, career exploration; FWS, future work self; CI, career indecision; CP, career planning.

Harman's single factor method was used to extract 5 factors with eigenvalues greater than 1 without rotation, explaining 58.298% of the total variation with the first principal component accounting for 20.867% of the total loading. There was no significant loading and no serious common method deviation problem.

**Figure 1:**
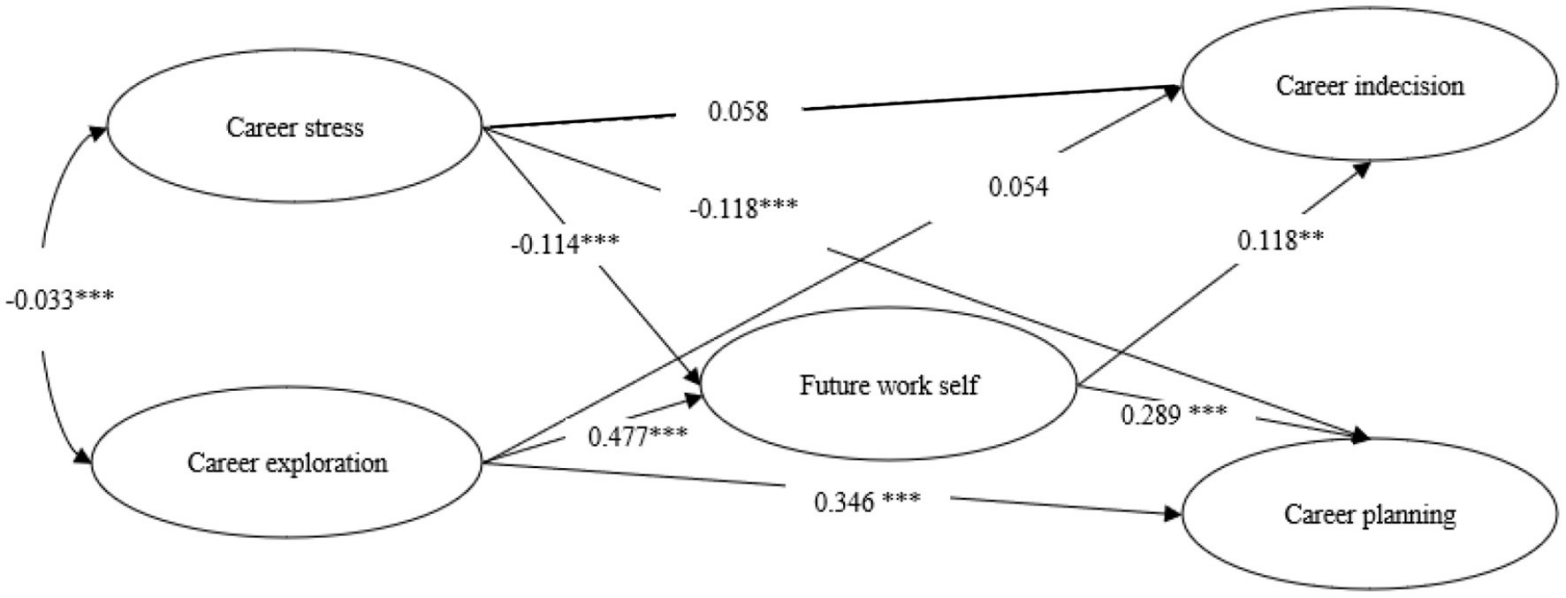
The meditation model with standardized coefficients. **p < 0.01, ***p < 0.001.

The following results transpired from the model as shown in Figure 1. Career stress negatively predicted both future work self (*B* = −0.107, β= −0.114, *p* < 0.001) and career planning (*B* = −0.165, β= −0.118, *p* < 0.001) while its direct effect on career indecision was not significant (*B* = 0.099, β = 0.058, *p* = 0.075). Meanwhile, future work self negatively predicted both career indecision (*B* = 0.215, β = 0.118, *p* < 0.01), and career planning (*B* = 0.429, β = 0.289, *p* < 0.001). Thus, future work self played a complete mediating role in the effect of career stress on career indecision (*B* = −0.023, β = −0.013, 95% CI = −0.054 to −0.005, *p* = 0.005), but played a partial mediating role in the influence of career stress on career planning (*B* = −0.046, β = −0.033, 95% CI = −0.079 to −0.017, *p* = 0.004).

Career exploration positively predicted both future work self (*B* = 0.456, β = 0.477, *p* < 0.001), and career planning (*B* = 0.491, β = 0.346, *p* < 0.001). While its direct effect on career indecision was not significant (*B* = 0.094, β = 0.054, *p* = 0.164). Similarly, the future work self played a complete mediating role in the effect of career exploration on the career indecision (*B* = 0.098, β = 0.056, 95% CI = 0.026 to 0.181, *p* = 0.006), and partially mediating role in the effect of career exploration on career planning (*B* = 0.196, β = 0.138, 95% CI = 0.142 to 0.262, *p* = 0.001).

## Discussion

### Theoretical implications

The study provides several key theoretical implications: first, while most previous studies explore either the effect of context factor or the effect of individual initiative factor on occupational behaviors (Choi et al., 2011; Cheung and Arnold, 2014; Cheung and Jin, 2016), this study considered the combination effect of contextual (career stress) and individual initiative factors (career exploration) from a composite perspective, enriching the current discourse on career construction theory. Results show that both career stress and career exploration have significant effects on the future work self, which is consistent with hypothesis 1 and hypothesis 2. Kao et al. (2020) studied the supportive context of future work self, but there is still a lack of research on the impact of career stress (as obstructive context) on future work self. Lee et al. (2013) and You and Yoo (2021) have demonstrated the impact of career stress on life satisfaction and depression from the perspective of general psychology. Therefore, our study would be supplement to verify the negative impact of career stress on future work self from the perspective of occupational psychology. The positive effect of career exploration on future work self has been proved by Guan et al. (2017) and Xiao et al. (2021), our study also supports this conclusion. When examining joint effects, we found that career exploration plays a greater role; implying the importance and effectiveness of individual initiative in dealing with stressful situations (Arbona et al., 2021). This also coincides with career construction theory's emphasis on the exertion of individual initiative to construct one's own career.

Second, it is interesting to note that by examining the mediating role of the future work self, results indicate a double-sided effect. Although the study confirms the mediating effect of the future work self and verifies that career stress and career exploration influence both career indecision and career planning through future work self (h5 and h6 were verified), a double-sided is nonetheless seen. On one hand, the salience of the future work self has positive effect on career planning, coinciding with our hypothesis 3. On the other hand, results show that the salience of future work self also has positive effect on career indecision, which does not coincide with our hypothesis 4. Most of the existing studies focus on the positive effects of future work self, such as improving college students' career adaptability (Guan et al., 2017; Chan and Chan, 2021), increasing proactive career behavior (Taber and Blankemeyer, 2015) and job hunting behavior (Kao et al., 2020), few studies have considered its negative effects. Our study provides a new perspective on the exploration of the effects of the future work self on Chinese college students—they face many external pressures on their career behavior because of the overarching Confucian culture, including the orthodox and mainstream views of society, filial piety, comments from relatives and friends and so on. Ouyang et al. (2016) found it was important for Chinese students to obtain social approval when making career choice and they always hesitated to challenge vocational options conferred by others. Therefore, we suspect that these external pressures may ultimately contribute to this result. When students' future work selves conflict with external requirements, they are more likely to face indecision. Since previous studies focus more on the intrinsic motivation of individuals, future studies should pay more attention to the influence of other external pressures on Chinese college students' vocational behaviors.

Third, in examining the effects of career exploration on career planning and career indecision, we get some supplementary results. Career exploration can promote career planning, but can also exacerbate career indecision through the mediating effect of future work self. Previous studies have found that career exploration may improve self-efficacy or confidence in career decision making (Cheung and Arnold, 2014; Cheung and Jin, 2016; Lent et al., 2016), but does not necessarily reduce career indecision (Jiang et al., 2019). This study identifies how career exploration leads to career indecision. As mentioned above, a salient future work self may lead to career indecision, especially if there is a lack of career support. Jiang et al. (2019) pointed out that the level of career support and decision-making guidance were also important factors in the process of career decision-making (Lent et al., 1994; Savickas, 2002), which may either strengthen or weaken the influence of career exploration on career decision-making. Since most of the Chinese college students lack the basic knowledge related to career, as well as the necessary support and guidance in university during career exploration, they may face the dilemma of more exploration leading to more confusion.

### Practical implications

These results of this study are expected to provide a reference for counselors and students.

First, students should make full use of their subjective initiative to deal with stressful situations when making career planning and career decision. Career exploration is one way to exert initiative, which is helpful for college students to form cognitions of themselves and the employed world, to learn career-related knowledge and skills, and to accumulate career-related resources (Blustein, 1989; Zikic and Hall, 2009). College students should actively explore themselves and environment (Stumpf et al., 1983). In terms of self-exploration, they should learn to contemplate their past, reflect on how to integrate their past with future career, take career courses, and use assessment tools under the guidance of a consultant to clarify self-understanding. In terms of environment exploration, they should participate in more part-time jobs, go to various career orientation programs and get more career information on the labor market and job opportunities by communicating with graduates and knowledgeable others. Overall, Career exploration should be purposeful, and should answer students' questions of what they want to do in the future to develop a salient future work self.

Second, students shouldn't blindly explore when doing career exploration, and counselors should offer necessary guidance. Since Chinese students may become increasingly indecisive during career exploration, it's necessary to pay attention to other factors which will influence the effects of career exploration, such as the level of career support and decision-making guidance (Lent et al., 1994; Savickas, 2002). Counselors should offer necessary guidance as most Chinese students lack the basic knowledge of career exploration. During career exploration, counselors should inform students with basic knowledge of career exploration, instruct students to use relevant self-exploration tools and provide more opportunities and access to career information including professional lectures and salons. All these may help students refine their effective career exploration skills. In addition, counselors should also provide necessary intervention when students encounter difficulties in their career exploration.

Third, counselors and students should beware of the double-sided effects of the future work self. Since it is positively related to career planning, a salient future work self may help students set goals and inspire actions, and is therefore necessary for them to own. However, a salient future work self can also induce career indecision, especially when it conflicts with external factors. Therefore, two suggestions are offered: counselors should analyze the salience of students' future work selves through assessment tools or interviews in the process of career guidance. For students with low salience of future work selves, counselors should provide apt interventions. For students with high salience of future work selves, counselors should help them be aware of the difficulties in achieving future work selves and find resolutions. It is necessary to intervene with students who are facing greater conflict issues. Meanwhile, students should comprehensively consider the influence of internal and external factors. In establishing future work selves, they should clarify their own ideas and communicate well with their families, teachers, important others to reduce possible conflicts. Through this, they can enhance confidence in realizing future working selves and avoid career indecision.

### Limitations and future research

The limitations of this study are as follows: first, a cross-sectional study design was used in this study, which made the specific causal effects of career stress and career exploration on career planning and career indecision unaccountable. Although we measured variables at different time points, we were unable to measure all variables at each time point. Therefore, we could not establish an absolute causal relationship between different measures. Although career planning and career indecision may appear to be the result of career stress and career exploration, these can also be viewed as causes. Research has also shown that career indecision can lead to cognitive and physical anxiety (Miller and Rottinghaus, 2014), and career indecision can also be regarded as a source of career stress (Choi et al., 2011). In addition, career decision-making process and career exploration process interact with each other. While career exploration promotes the progress of career decision-making (Lent et al., 2016, 2017), career indecision is also a cause of career exploration (Vignoli, 2015). In the future, we can adopt a longitudinal research design to further understand the dynamic relationship between these variables.

Secondly, this study is mainly conducted from the perspective of college students themselves, but does not include the role of other external factors such as parents' support, teachers' support, social mainstream views, and so on. In the Chinese cultural context, these external factors may have an important impact on the students' career behaviors. Zhou et al. (2012) found that traditional Chinese values (e.g., career as a way to repay parents and sustain family wellbeing) are still prominent in Chinese students' conception of career. (Leung et al., 2011) observed on the career goals of individuals from a collectivistic culture, and found that the goals and values of the group are internalized to a point where the distinction between personal and collective goals are hard to draw. Therefore, even if an individual has a clear future work self, without the support of these external factors, they may still encounter difficulties such as career indecision. Future studies can further study the effects of these external pressures on Chinese students. Moreover, considering the differences between Chinese and Western cultures and the unexpected impact of future work selves on Chinese students in this study, future studies can also conduct a comparative study on the influence of future work selves of Chinese and Western students.

## Data availability statement

The raw data supporting the conclusions of this article will be made available by the authors, without undue reservation.

## Ethics statement

Ethical review and approval was not required for the study on human participants in accordance with the local legislation and institutional requirements. All subjects gave written informed consent in accordance with the Declaration of Helsinki.

## Author contributions

ZZ wrote the manuscript under the guidance of XY. XY contributed to the study design and critical revisions and was in charge of manuscript proofreading and checking. XL was in charge of data analysis. All authors contributed to the article and approved the submitted version.

## Conflict of interest

The authors declare that the research was conducted in the absence of any commercial or financial relationships that could be construed as a potential conflict of interest.

## Publisher's note

All claims expressed in this article are solely those of the authors and do not necessarily represent those of their affiliated organizations, or those of the publisher, the editors and the reviewers. Any product that may be evaluated in this article, or claim that may be made by its manufacturer, is not guaranteed or endorsed by the publisher.
